# iPSC-Derived Glioblastoma Cells Have Enhanced Stemness Wnt/β-Catenin Activity Which Is Negatively Regulated by Wnt Antagonist sFRP4

**DOI:** 10.3390/cancers15143622

**Published:** 2023-07-14

**Authors:** Ishmat Ara Yasmin, Arun Dharmarajan, Sudha Warrier

**Affiliations:** 1 Division of Cancer Stem Cells and Cardiovascular Regeneration, Manipal Institute of Regenerative Medicine, Manipal Academy of Higher Education (MAHE), Bangalore 560 065, India; ishmat.yasmin@learner.manipal.edu; 2 Department of Biomedical Sciences, Faculty of Biomedical Sciences and Technology, Sri Ramachandra Institute of Higher Education and Research, Chennai 600 116, India; 3 School of Human Sciences, Faculty of Life and Physical Sciences, The University of Western Australia, Perth, WA 6009, Australia; 4 Curtin Medical School, Curtin University, Perth, WA 6102, Australia; 5 Cuor Stem Cellutions Pvt Ltd., Manipal Institute of Regenerative Medicine, Manipal Academy of Higher Education (MAHE), Bangalore 560 065, India

**Keywords:** amniotic-membrane mesenchymal stem cells, induced pluripotent stem cells, cancer stem cells, glioblastoma multiforme, glioblastoma stem cells, Wnt/β-catenin pathway, secreted frizzled-related protein 4

## Abstract

**Simple Summary:**

Cancer is the second most complex disease after cardiovascular disease. Glioblastoma multiforme (GBM) is a heterogeneous tumor in which the small population of cancer stem cells (CSCs) confers tumors with stemness, relapse and chemotherapeutic resistance. The complete knowledge of the key factors driving CSCs is unclear. This emphasizes the need of the CSC model to understand molecular mechanisms. However, to date, a rapid and readily available in vitro model of GBM of human origin has not been available. In this study, we demonstrated the generation of induced pluripotent stem cells (iPSCs) from a perinatal source amniotic membrane-derived mesenchymal stem cell (AMMSC). A rapid iPSC-derived GBM model exhibiting upregulated canonical Wnt/β-catenin activity was then established. Further, the upregulated Wnt activity could be inhibited by the Wnt antagonist sFRP4. Our study will provide a rapid and easy cell-based platform for understanding the underlying mechanisms of GBM progression and help in assessing chemotherapeutic drugs.

**Abstract:**

Growing evidence indicates that cancer stem cells (CSCs) endow the tumor with stem-like properties. Recently, induced pluripotent stem cells (iPSCs) have gained increased attention because of their easy derivation and availability and their potential to differentiate into any cell type. A CSC model derived from iPSCs of human origin would help understand the driving force of tumor initiation and early progression. We report the efficient generation of feeder-free SSEA4, TRA-1-60 and TRA-1-81 positive iPSCs from amniotic membrane-derived mesenchymal stem cells (AMMSCs), which successfully differentiated into three germ layers. We then developed human iPSC-derived glioblastoma multiforme (GBM) model using conditioned media (CM) from U87MG cell line and CSCs derived from U87MG, which confer iPSCs with GBM and GSC-like phenotypes within five days. Both cell types overexpress MGMT and GLI2, but only GSCs overexpress CD133, CD44, ABCG2 and ABCC2. We also observed overexpression of LEF1 and β-catenin in both cell types. Down-regulation of Wnt antagonist secreted frizzled-related protein 4 (sFRP4) in GBM and GSCs, indicating activation of the Wnt/β-catenin pathway, which could be involved in the conversion of iPSCs to CSCs. From future perspectives, our study will help in the creation of a rapid cell-based platform for understanding the complexity of GBM.

## 1. Introduction

The generation of cancer stem cell (CSC) models from induced pluripotent stem cells (iPSCs) offered an attractive opportunity to study and determine the underlying mechanisms of disease progression and the origin of CSCs [1]. iPSCs exhibit characteristic features of stemness and pluripotency like embryonic stem cells (ESCs). It was in 2006 that Yamanaka’s group reported the breakthrough discovery of reprogramming mouse fibroblasts into mouse iPSCs [2]. In late 2007, human iPSCs were generated from human fibroblasts [3,4]. Since then, various strategies have been employed to generate mouse iPSCs and human iPSCs from various sources. Human iPSCs appear to be the epitome of disease models as they surmount the ethical and immunological concerns raised by human ESCs [5]. The generation of iPSCs from a patient’s cells circumvents tissue rejection during transplantation [2]. Therefore, it has drawn significant attention in autologous transplantation. The generation of integration-free iPSCs holds enormous advantages in cell-based therapies, as it is considered ‘safe’ from any other mutation [6], and it is routinely produced at the Center for iPS Cell Research and Application (CiRA) at Kyoto University, Japan [7]. As animal models or cell culture systems do not recapitulate the human system, iPSCs have ushered in its utility by delivering human-relevant cell sources. iPSCs overcome the problems that arose from differences between human and animal systems [8]. Thus, iPSCs have been a promising tool to understand etiology, treat various incurable diseases, and screen drugs.

Glioblastoma multiforme (GBM) is a heterogeneous tumor which has small populations of CSCs at its apex, which acts as a key driver for chemotherapeutic drug resistance [9]. The origin of CSCs has an impact in clinical stages as residual CSCs, which escape from chemotherapy, may lead to relapse [10]. CSCs constitute 0.01–2% of the tumor [11]. CSCs share common transcription factors with ESCs like OCT3/4 [12], SOX2 [13], KLF4 [11] and c-Myc [14]. Investigators postulated several hypotheses on the origin of CSCs and their driving force. The self-renewal property of CSCs is regulated by crosstalk between the Wnt/β-catenin pathway, Notch pathway and Hedgehog pathway, which are also shared by normal stem cells [11]. Although both CSCs and normal stem cells share a common signal transduction pathway for their proliferation and differentiation, dysregulation of these pathways results in uncontrolled growth of CSCs and leads to tumorigenesis [11,15]. However, the principal pathway that regulates CSCs is yet to be explored. Inhibitors of Notch, Hedgehog and receptor tyrosine kinase pathways and inhibitors of receptors of the epidermal growth factor and the vascular endothelial growth factor targeting glioma stem cells have shown efficacy in preclinical trials. However, targeting glioma stem cells via Wnt/β-catenin is difficult, as the Wnt pathway regulates normal physiological processes as well [16,17].

As CSCs act as a seed for tumorigenesis, it is particularly important to know how stemness properties convert into tumorigenesis and which are the principal molecules/pathways responsible for this. Establishing the iPSC-based CSC model would surmount the numerous challenges in understanding the molecular mechanisms underlying various stages of cancer development. Although xenotransplantation is the most acceptable model for studying human malignancies, certain caveats are overlooked, such as the response of the human immune system and other CSC properties [10]. Species differences also prevent complete recapitulation of the underlying mechanism of disease progression [18]. To date, there is no single experimental in vitro model of human or mouse origin which could recapitulate all the biological complex features of glioblastoma.

Although liver, lung, and breast CSC models from iPSCs have been reported using conditioned media, there is no model available for iPSC-derived glioblastoma using conditioned media (CM) [1,19,20,21,22]. Hwang et al. 2020 demonstrated the generation of an organoid model from iPSCs which mimics GBM features [23]. In addition, Azzarelli et al. 2021 described an approach towards understanding the fate of glioma stem cells (GSCs) by co-culturing them with human brain organoids [24]. Although iPSC-derived glioblastoma models have been developed, these models focused only on the disease state but not on the origin of CSCs or the principal factors which regulate CSCs.

There are various signal transduction pathways that play a significant role in the maintenance of CSCs, but the principal pathway that regulates stemness remains elusive. A previous study also reported that Wnt antagonists are epigenetically silenced in GBM [25]. Furthermore, it has been reported that the Wnt antagonist secreted frizzled-related protein 4 (sFRP4) reduces the stemness of glioblastoma by its netrin-like domain [26]. Considering this evidence and the research gap identified, we hypothesized that the Wnt pathway could be a determining mechanism in maintaining glioma stemness.

In the present study, we demonstrate a rapid and efficient generation of an elegant glioblastoma and glioblastoma stem cells (GSCs) model from iPSCs derived from amniotic membrane-derived mesenchymal stem cells (AMMSCs). This diseased model mimics the molecular markers of glioblastoma stem cells. The study also delineates that the Wnt/β-catenin pathway is the determining factor for driving stemness into tumorigenicity. We anticipate that our study will shed light on the underlying molecular mechanism of initiation of GSCs which would enhance in vitro disease modelling and the development of the new therapeutic intervention.

## 2. Methods

### 2.1. Isolation, Cell Culture and Maintenance

Mesenchymal stem cells (MSCs) were isolated from an amniotic membrane (AM) of a human placenta via enzymatic digestion as described in Warrier et al. 2012 [27]. AMMSCs were cultured and maintained in Dulbecco’s Modified Eagle Medium (DMEM) with 10% fetal bovine serum (FBS) and 1X antibiotic solution in a humid incubator with 5% CO_2_ at 37 °C. Details of materials are available in Supplementary Table S1.

### 2.2. Reprogramming of AMMSCs, Generation of Induced Pluripotent Stem Cells (iPSCs), Maintenance and Cryopreservation

AMMSCs were reprogrammed with Shinya Yamanaka’s factors pCXLE-hOCT3/4-shp53-F, pCXLE-hSK, and pCXLE-hUL using Lipofectamine^TM^3000 transfection reagent. iPSCs were generated and maintained in pluripotent stem cell media composed of Knockout ^TM^ DMEM 1X, optimized for ES cells, 20% Knockout ^TM^ serum replacement, 2 mM GlutaMAX ^TM^ supplement, 1X MEM non-essential amino acids solution (NEAA), 0.1 mM 2-mercaptoethanol, 10 ng/mL human recombinant fibroblast growth factor 2 (FGF2) protein, and 1X antibiotic solution [3,28]. Feeder-free iPSC colonies were maintained on Matrigel, expanded, cryopreserved, and thawed to generate stocks for all subsequent experiments. iPSC colonies were visualized using Nikon Eclipse TE2000-U inverted phase contrast microscope.

### 2.3. Lipid Body-Associated Retinyl Ester Fluorescence Imaging

To detect blue fluorescence from lipid bodies of iPSC, colonies were visualized at 450–500 nm using the Nikon Eclipse TE2000-U inverted fluorescence microscope (Nikon, Tokyo, Japan) [29].

### 2.4. Alkaline Phosphatase Staining

iPSC colonies were picked with the help of p200 and transferred to DMEM/F12 media in a 4-well plate (Corning Inc., Corning, NY, USA). Colonies were then stained for alkaline phosphatase using 1X Alkaline phosphatase live stain according to the manufacturer’s instructions. The colonies were visualized using the Nikon Eclipse TE2000-U inverted fluorescence phase contrast microscope (Nikon, Tokyo, Japan). Colonies showing green fluorescence as positive AP expression were then transferred to pluripotent stem cell media. Colonies were either directly cryopreserved and thawed whenever required or manually dissected with the help of p200 and expanded [30].

### 2.5. RNA Extraction, cDNA Synthesis, Quantitative Real-Time PCR (qRT-PCR)

The total RNA was isolated by RNAiso Plus, and cDNA synthesis was carried out by the PrimeScript 1st strand cDNA synthesis kit. The mRNA expressions were quantified by quantitative real-time PCR using TB Green^®^ Premix Ex Taq™ II in a QuantStudio™ 5 real-time PCR Systems (Applied Biosystems, Waltham, MA, USA) as described in Yasmin et al. 2021 [26]. Primers were synthesized from Bioserve, Biotechnologies Pvt Ltd., India and Sigma-Aldrich, St. Louis, MO, USA. GAPDH was used as an endogenous control. Primer sequences were designed using Primer-BLAST (NCBI, NLM, NIH, US) and mentioned in Table 1.

### 2.6. Immunocytochemistry

Immunocytochemical analysis was performed as described by Yasmin et al., 2021 [26]. For the detection of cell surface antigens, SSEA4, TRA-1-60, TRA-1-81, CD133, and CD44, cells were processed without permeabilization. Cells were stained with mouse anti-Oct-4 antibody (1:500), purified mouse anti-Sox2 antibody clone 030-678 (1:200), mouse anti-stage-specific embryonic antigen-4 (SSEA-4) antibody, clone MC-813-70 (1:500), purified mouse anti-human TRA-1-60 antigen (1:200), mouse anti-TRA-1-81 (1:500), rabbit anti-vimentin (1:500), rabbit paired box gene 6 (PAX6) (1:200), rabbit Nkx2.5 polyclonal antibody (1:300), mouse CD133 monoclonal antibody FITC, Clone: EMK08, eBioscience^TM^ (1:200), mouse CD44 monoclonal antibody (IM7), FITC, eBioscience^TM^ (1:200), and rabbit β-catenin polyclonal antibody (1:500). Next, cells were incubated with goat anti-mouse IgG (H+L) highly cross-adsorbed secondary antibody, Alexa Fluor^TM^ 594 (1:1000), goat anti-rabbit IgG(H+L) highly cross-adsorbed secondary antibody, Alexa Fluor^TM^ 488, goat anti-rabbit IgG(H+L) cross-adsorbed secondary antibody, Alexa Fluor ^TM^ 594. The nuclei were counterstained with 10 μg/mL of Hoechst 33342, trihydrochloride, trihydrate solution in water or 4’,6-Diamidino-2-phenylindole dihydrochloride (DAPI). Details of antibodies are available in Supplementary Table S2. All fluorescence cells were observed using the Nikon Eclipse TE2000-U inverted fluorescence phase contrast microscope (Nikon, Tokyo, Japan) and Olympus IX73 (Olympus, Tokyo, Japan). Photographs were recorded and processed using Q-Capture Pro 6 and 7 and ImageJ 1.51j8 (NIH, Bethesda, MD, USA) software, respectively.

### 2.7. In Vitro Differentiation of iPSCs

#### 2.7.1. Ectoderm Differentiation

iPSC colonies were differentiated into ectoderm lineage according to Miltenyi manufacturer’s instructions with few modifications [31,32]. For ectoderm differentiation, iPSC colonies were thawed and allowed to grow for 2 more days. On day 2, iPSCs were grown in pluripotent stem cell media with 10 μM SB431542 hydrate. On day 6, for neuroectoderm lineage differentiation, SB431542 media was removed and replaced with DMEM/F-12, 1X N-2 supplement, 1X B-27^TM^ supplement, serum-free for 24 h. On day 7, fresh media was added. For lineage marker analysis, on day 9, cells were analyzed for PAX6 expressions by immunocytochemistry.

#### 2.7.2. Mesoderm Differentiation

iPSC colonies were differentiated into mesoderm using different protocols with few modifications [33,34,35,36,37]. For mesoderm differentiation, once iPSC colonies reach 60% confluency, 8–10 colonies were transferred to a matrigel-coated 96-well plate (Corning Inc., Corning, NY, USA) and allowed to grow for 1 more day. On day 1, iPSCs were allowed to grow for 24 h in RPMI 1640, 1X supplemented with 6 μM CHIR99021. The next day, CHIR99021 was removed. On day 4, cells were induced with RPMI 1640, 10 μM IWR-1, 213 μg/mL ascorbic acid (AA) and allowed to grow for four more days. On day 8, media was replaced with RPMI 1640, 1X B-27^TM^ supplement, 213 μg/mL AA, 20 μg/mL insulin, 1X insulin transferrin selenite (ITS), and fresh media was added on day 10. On day 13, the media was replaced with 10 μM SB431542. On day 15, cells were analyzed for Nkx2.5 expression by immunocytochemistry.

#### 2.7.3. Endoderm Differentiation

iPSC colonies were differentiated into endoderm lineage using different protocols with few modifications [38,39,40,41,42,43,44,45,46]. For endoderm differentiation, once iPSC colonies reach 60% confluency, 8–10 colonies were transferred to a matrigel-coated 96-well plate (Corning Inc., Corning, NY, USA) and allowed to grow for 2 more days. On day 2, pluripotent stem cell media was replaced with RPMI1640, 1X supplemented with 75 ng/mL Wnt 3a, 3 μM CHIR99021, 100 ng/mL Activin A and 2% Knockout ^TM^ serum replacement. On day 6, after 96 h, old media was replaced with fresh RPMI 1640, 2% Knockout ^TM^ serum replacement and 100 ng/mL Activin A for 24 h. On day 7, pancreatic lineage differentiation was induced by adding fresh media RPMI 1640, 10 μM SB431542 hydrate, 10 mM Nicotinamide, 2 μM retinoic acid and 0.5 μg/mL epidermal growth factor (EGF).

##### Dithizone (DTZ) Staining

DTZ staining was performed to stain pancreatic β-cells as described in Shiroi et al., 2002 [47]. After staining, cells were observed using the Nikon Eclipse TE2000-U inverted phase contrast microscope (Nikon, Tokyo, Japan).

### 2.8. Cell Culture

Human glioblastoma multiforme (GBM) cell line U87MG was procured from the National Centre for Cell Science (NCCS), Pune, India. The cell line was cultured and maintained as described by Yasmin et al., 2021 [26].

### 2.9. Enrichment and Characterization of Cancer Stem Cells (CSCs)

CSCs were enriched from U87MG as described by Yasmin et al., 2021 [26]. The cells were characterized for CSC specific markers CD133, CD44, ABCG2 and ABCC2 by qRT-PCR, immunocytochemical analysis and flow cytometry analysis.

### 2.10. Flow Cytometry

U87MG monolayer and CSCs were trypsinized, and cell pellets were fixed with 4% paraformaldehyde for 10 min at room temperature and washed twice with PBS, followed by blocking with 2% bovine serum albumin (BSA) for 30 min at room temperature. Cells were then stained with rat anti-human/mouse CD44 monoclonal antibody (IM7), FITC (1:100) overnight at 4 °C, followed by washing twice with PBS. FITC rat IgG2b, κ isotype control antibody (1:100) was used as isotype control. Data was acquired for CD44 by BD FACS LSRII (Becton and Dickinson, San Diego, CA, USA) and BD FACSDiva 8.0.1 software. Data was analyzed by FCS Express 6 software [48].

### 2.11. Transient Transfections

U87MG cells were transiently transfected with expression vector EGFP-N1 with sFRP4 inserts as reported previously [26]. Overexpression of sFRP4 in cell lysates was confirmed and secretion of sFRP4 in conditioned media was validated by indirect ELISA.

### 2.12. Conditioned Media (CM)

CM was harvested from monolayer, CSCs enriched from U87MG and sFRP4 overexpressed U87MG as reported previously [26].

### 2.13. Indirect Enzyme-Linked Immunosorbent Assay (ELISA)

Indirect ELISA was performed as described in Han et al. 2022 with few modifications [49]. Briefly, cell lysates were extracted from control and sFRP4-EGFP transfected U87MG using RIPA buffer, 1X protease inhibitor cocktail, and 1 mM phenylmethanesulfonylfluoride (PMSF). CM was collected from control and sFRP4-EGFP transfected U87MG cells as reported previously [26]. A 96-well plate was coated with 20 μg/mL of cell lysates and CM overnight at 4 °C followed by blocking with 1% BSA for 2 h in room temperature and incubation with rabbit sFRP4 polyclonal antibody (1:500) overnight at 4 °C. Goat anti-rabbit IgG(H+L) secondary antibody, HRP (1:500) was then added to the wells and incubated for 2 h in room temperature followed by the addition of substrate 3,3′,5,5′-tetramethylbenzidine (TMB) and incubation for 30 min. Each of the steps were followed by washing with PBS. Reaction was stopped with 1 M H_2_SO_4_ and sFRP4 expression in cell lysates, and CM were quantified by recording absorbance at 450 nm using an EnSight^TM^ Multimode plate reader (Perkin Elmer, Waltham, MA, USA).

### 2.14. Conversion of iPSCs to Glioblastoma Cells, Glioblastoma Stem Cells and sFRP4 Overexpressed Glioblastoma Cells

The conversion was carried out as described in Afify et al. 2019 with few modifications [1]. Briefly, harvested CM was used to convert iPSCs into glioblastoma multiforme models. On day 0, iPSCs were thawed on a matrigel-coated plate. iPSC colonies were then allowed to grow for 24 h. The next day, an equal number of colonies were seeded in matrigel-coated 4 wells of a 24-well plate (Corning Inc., Corning, NY, USA). Pluripotent stem cell media was replaced with 50% harvested CM (1:1, iPSC media: CM) without human recombinant fibroblast growth factor 2 (FGF2) protein. Control iPSCs were maintained in complete pluripotent stem cell media. Every day, the media was replaced with fresh media. Cells were kept for conversion for five days.

### 2.15. Co-Immunostaining

iPSC-derived glioblastoma stem cells were equally seeded on a matrigel-coated plate and allowed to grow for 24 h. A total of 500 pg/mL of recombinant human sFRP4 protein was added in serum-free media and incubated for 24 h. Co-immunostaining was performed to double stain nuclear localization of β-catenin and cell surface marker CD44 [48].

### 2.16. Statistical Analysis

Statistical analysis was carried out by one-way analysis of variance (ANOVA) followed by Tukey’s multiple comparison test and student’s *t*-test using GraphPad Prism 5 software (San Diego, CA, USA). For significant differences between control and treated conditions, * *p* < 0.05, ** *p* < 0.001, *** *p* < 0.0001 was taken into consideration.

## 3. Results

### 3.1. Reprogramming and Characterization of AMMSC-Derived iPSCs

**Figure 1:**
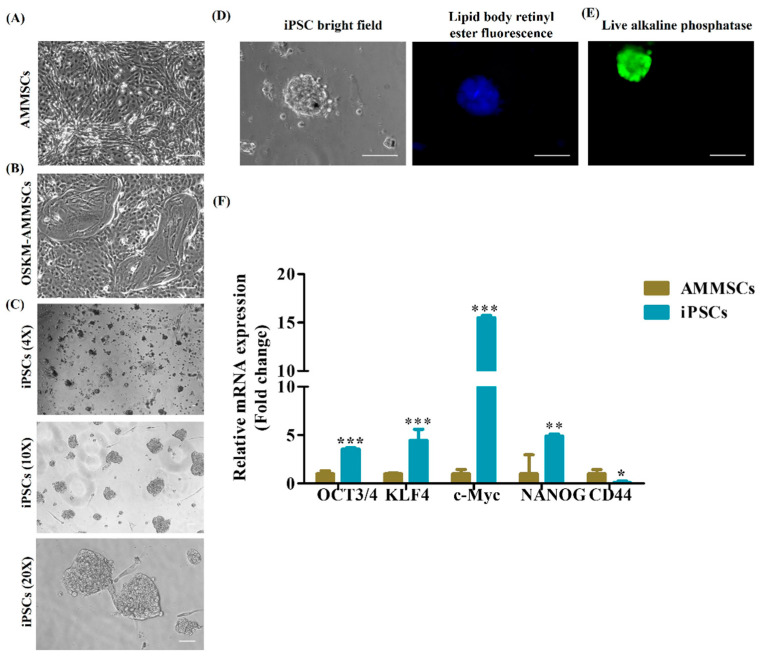
Generation of induced pluripotent stem cells (iPSCs) and its characterization. (**A**) Photomicrographic representation of amniotic membrane mesenchymal stem cells (AMMSCs) isolated from human placenta, scale bar = 100 μm. (**B**) Photomicrographic representation of AMMSCs reprogrammed with Yamanaka factors OSKM plasmid, scale bar = 100 μm. (**C**) Photomicrographic representation of iPSCs generated from reprogrammed AMMSCs at 4×, 10× and 20×, scale bar = 100 μm. (**D**) Representation of blue fluorescence of cytoplasmic lipid bodies of iPSCs using DAPI filter and captured under fluorescence microscope, scale bar = 100 μm. (**E**) Image of live alkaline phosphatase positive iPSCs, scale bar = 100 μm. (**F**) qRT-PCR analysis of OCT3/4, KLF4, c-Myc, NANOG, and CD44 in iPSCs and in their parental AMMSCs. Data are shown as mean ± SEM. * *p* < 0.05, ** *p* < 0.001, *** *p* < 0.0001, *n* = 3.

AMMSCs (Figure 1A) were transfected with Yamanaka’s factors pCXLE-hOCT3/4-shp53-F, pCXLE-hSK, and pCXLE-Hul. After transfection of AMMSCs, they underwent visible morphological changes, and reprogrammed cells acquired morphology that resembled a ‘hollow cavity’ (Figure 1B). Typical iPSC colonies with borders appeared from day 10–12 (Figure 1C), which clearly indicates successful reprogramming of AMMSCs. The iPSC colonies were then examined under a fluorescence microscope, and characteristic blue fluorescence of cytoplasmic lipid bodies was observed (Figure 1D). These lipids’ body-associated blue fluorescence is positively correlated with the pluripotent state of iPSCs and is inversely related to differentiation [29].

Next, iPSC colonies were evaluated for alkaline phosphatase (AP) expression. Measuring AP expression has emerged as an initial benchmark evaluation for iPSCs. AP production is directly proportional to pluripotency and determines cellular differentiation potential [50]. Live monitoring of iPSCs by AP staining confirmed elevated expression of phenotype marker AP (Figure 1E) and confirmed its undifferentiated state. AP positive colonies were picked up, and these AP positive colonies were cryopreserved and thawed whenever required for the next experiments.

**Figure 2:**
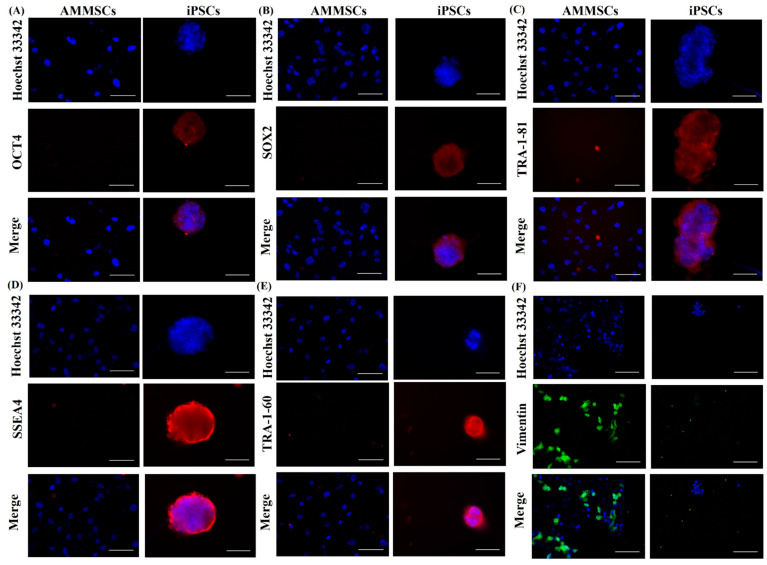
Immunocytochemical staining for iPSCs specific markers. (**A**) OCT4, (**B**) SOX2, (**C**) TRA-1-81, (**D**) SSEA4, (**E**) TRA-1-60 and mesenchymal stem cell specific marker (**F**) vimentin, scale bar = 100 μm.

After analyzing phenotypic markers to evaluate the iPSCs, we also measured the gene expression of ESC-specific markers OCT3/4, SOX2, NANOG [3] to identify bonafide iPSCs. iPSCs were confirmed by qPCR for elevated gene expression of reprogramming factors OCT3/4, KLF4, c-Myc, NANOG and decreased expression of MSC marker CD44 when compared to its parental cell AMMSCs (Figure 1F). In addition, immunofluorescence staining using anti-mouse Alexa fluor 594 secondary antibodies demonstrated elevated expression of OCT4, SOX2 in nucleus and cell surface antigens TRA-1-81, SSEA4, TRA-1-60 on iPSC colonies when compared to its parental cell AMMSCs (Figure 2). MSC marker vimentin, an intermediate filament, is also shown using anti-rabbit Alexa fluor 488 secondary antibody and was found to be decreased in iPSCs. Nuclei were visualized using Hoechst 33342 (Figure 2).

### 3.2. In Vitro Differentiation of iPSCs

**Figure 3:**
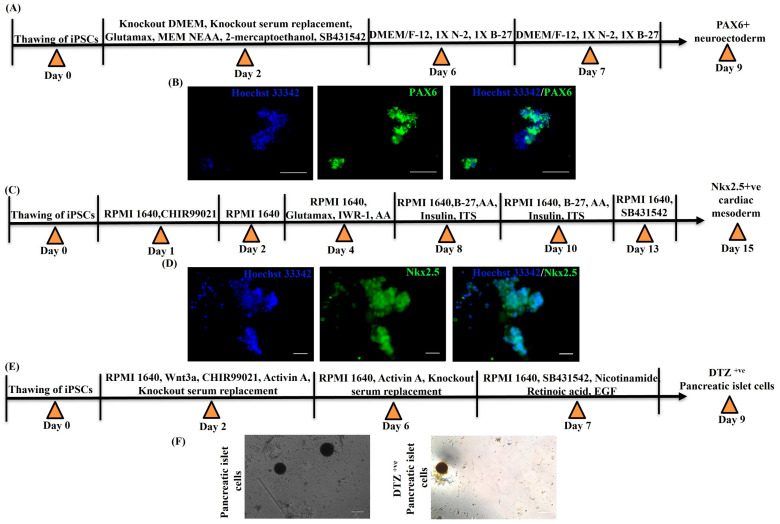
In vitro differentiation of iPSCs into ectoderm, mesoderm and endoderm. (**A**) Schematic representation of generation of the neuroectoderm from iPSCs. (**B**) Immunocytochemical staining of PAX6+ve cells in the neuroectoderm, scale bar = 100 μm. (**C**) Schematic representation of generation of Nkx2.5 +ve cardiac mesoderm cells from iPSCs. (**D**) Immunocytochemical staining of Nkx2.5 +ve cells in cardiac mesoderm, scale bar = 100 μm. (**E**) Schematic representation of generation of DTZ+ve pancreatic islet cells from iPSCs. (**F**) Photomicrographic representation of dithizone staining of pancreatic islet cells generated from iPSCs, scale bar = 100 μm.

Next, the iPSC colonies were confirmed for their differentiation ability into three germ layers. First, neuroectoderm was generated after sequential induction of iPSCs with small molecules and growth factors (Figure 3A). On day 9, neuroectoderm was stained for paired box transcription factor (PAX6), a characteristic marker of neuroectoderm, and immunocytochemical staining revealed that PAX6 positive neuroectoderm was generated from iPSCs (Figure 3B). This indicates that the iPSCs were successfully differentiated into ectoderm lineage.

Next, we differentiated iPSCs into cardiac mesoderm using a combination of agonist and antagonist of the Wnt pathway as well as small molecules (Figure 3C). On day 15, cardiac mesoderm was stained for Nkx2.5, a characteristic marker of cardiac mesoderm, and Nkx2.5 positive stained cells were visualized. Our study demonstrated differentiation of iPSC into Nkx2.5 positive efficient cardiac mesoderm (Figure 3D). This confirms that our generated iPSC can also successfully differentiate into mesoderm lineage.

Next, iPSC cells were differentiated into endoderm lineage followed by pancreatic progenitor differentiation using modulators of Wnt pathway, TGF-β pathway, casein kinase I (CKI), ROCK kinase pathways, etc. iPSC-derived pancreatic beta cells were generated by day 9 (Figure 3E). These pancreatic beta cells were stained using diphenyl thiocarbazone or dithizone (DTZ) staining. DTZ is used to differentially stain zinc-containing β cells of endocrine islet cells from acinar and ductal cells [51]. iPSC-derived pancreatic beta cells were successfully stained positive for DTZ (Figure 3F). All these observations confirmed the differentiation of iPSCs into three germ layers: ectoderm, mesoderm and endoderm.

### 3.3. Conversion of iPSCs into Glioblastoma Cells and Glioblastoma Stem Cells and Their Characterization

**Figure 4:**
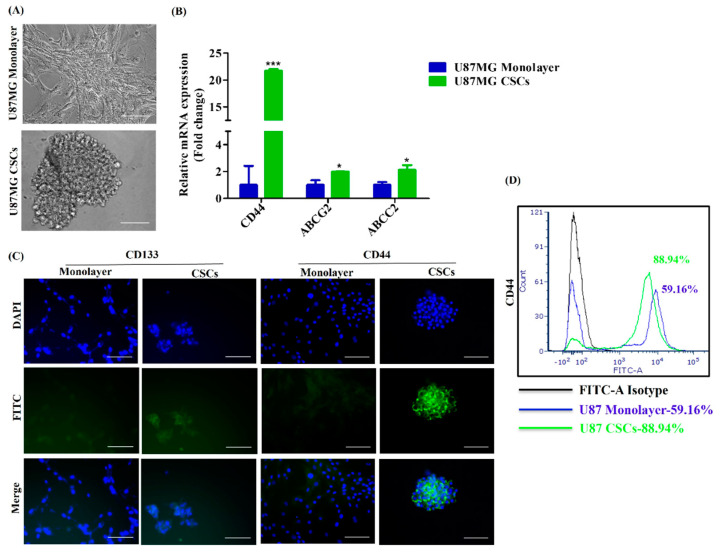
Enrichment and characterization of glioma stem cells from U87MG cells. (**A**) Photomicrographic representation of monolayer U87MG and cancer stem cells (CSCs) enriched from U87MG, scale bar = 100 μm. (**B**) qRT-PCR analysis of CD44, ABCG2 and ABCC2 in monolayer U87MG and U87MG CSCs. Data are shown as mean ± SD. * *p* < 0.05, *** *p* < 0.0001. (**C**) Immunocytochemical staining of CD133, CD44 in monolayer and CSCs of U87MG, scale bar = 100 μm. (**D**) Flow cytometry analysis of CD44 in monolayer and CSCs of U87MG.

**Figure 5:**
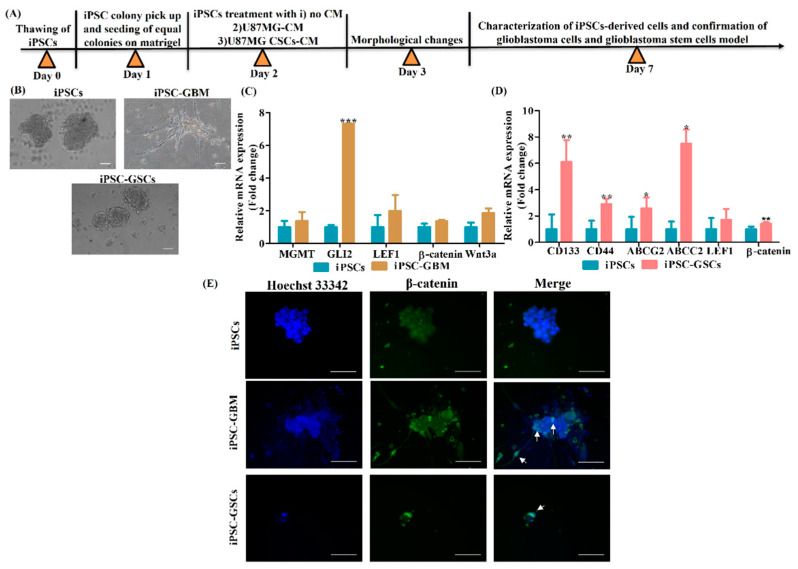
Generation of iPSC-derived glioblastoma cells (iPSC-GBM), glioblastoma stem cells (iPSC-GSCs) and their characterization. (**A**) Schematic representation of generation of iPSC-derived GBM and GSCs. (**B**) Photomicrographic representation of iPSCs, iPSC-GBM and iPSC-GSCs, scale bar = 100 μm. (**C**) qRT-PCR analysis of MGMT, GLI2, LEF1, β-catenin and Wnt3a expression in iPSC-GBM in comparison with iPSCs. (**D**) qRT-PCR analysis of CD133, CD44, ABCG2, ABCC2, LEF1 and β-catenin in iPSC-GSCs. Data are shown as mean ± SEM. * *p* < 0.05, ** *p* < 0.001, *** *p* < 0.0001, *n* = 3. (**E**) Immunocytochemical staining of β-catenin in iPSC-GBM and iPSC-GSCs, scale bar = 100 μm. Arrow indicates nuclear localization of β-catenin.

Next, we investigated the creation of a glioblastoma cell model from iPSC using U87MG conditioned media (CM). Therefore, we obtained U87MG monolayer (bulk GBM) CM as well as U87MG CSCs CM. Bulk GBM populations contain 0.01–2% CSCs [11]. Notably, CD133, CD44, ABCG2 and ABCC2 markers are lower in bulk GBM. Therefore, CSCs were enriched from the GBM cell line U87MG (Figure 4A) and were proven to overexpress CD44, ABCG2 and ABCC2, as confirmed by qPCR (Figure 4B). CSCs were observed to overexpress surface markers CD133 and CD44, as confirmed by immunocytochemical staining (Figure 4C). In addition, flow cytometry analysis also confirmed overexpression of CD44 in CSCs (88.94%) when compared to monolayer U87MG (59.16%) (Figure 4D). Next, iPSCs induced with a U87MG-conditioned medium (CM) (Figure 5A) are observed with morphological changes and neuron-like projections were observed (Figure 5B). iPSCs induced with U87MG CSCs CM (Figure 5A) did not undergo any morphological changes as CSCs are morphologically spherical (Figure 5B). iPSCs induced with U87MG-CM were found to overexpress the GBM-specific marker glioma-associated oncogene (GLI2) which confirmed the conversion of iPSCs to glioblastoma cells(iPSC-GBM). In addition, there was a tendency for upregulation of methyl guanine methyltransferase (MGMT) and Wnt canonical pathway regulators LEF1, β-catenin and Wnt3a (Figure 5C). iPSC induced with U87MG CSCs CM were found to overexpress CSC-specific markers CD133, CD44, ABCG2 and ABCC2, which confirmed these cells as iPSC-derived glioblastoma stem cells (iPSC-GSCs); these markers are more than monolayer U87MG, as observed (Figure 5D). Furthermore, there was a tendency for upregulation of LEF1 and enhanced β-catenin expression in iPSC-GSCs (Figure 5D). Furthermore, immunocytochemical staining demonstrated nuclear localization of β-catenin in iPSC-GBM and iPSC-GSCs compared to iPSCs, which shows cytoplasmic expression of β-catenin (Figure 5E). These observations indicate iPSCs were successfully converted into glioblastoma cells.

### 3.4. sFRP4 Reverses the Conversion of iPSC into Glioblastoma Cells and Inhibits iPSC-Derived GSCs

**Figure 6:**
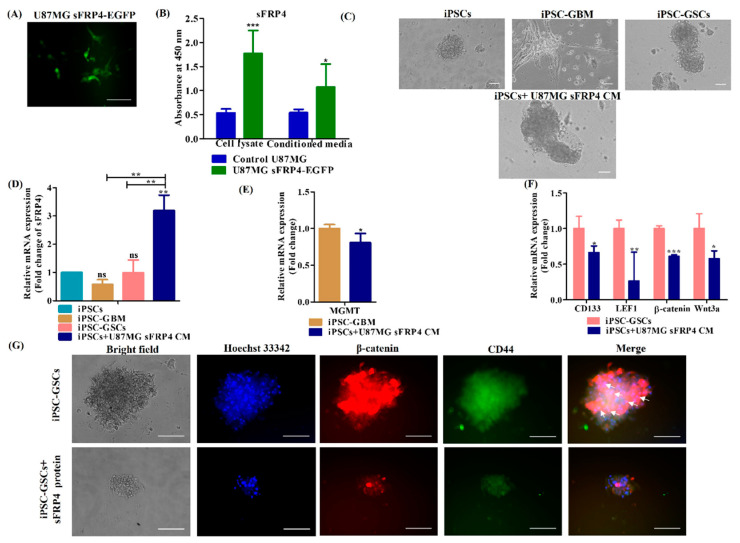
Effect of sFRP4 on the conversion of iPSCs into glioblastoma cells. (**A**) Photomicrographic representation of transfection efficiency by fluorescence microscopy, scale bar = 100 μm. (**B**) Quantitative analysis of overexpression of sFRP4 in cell lysates and validation of its secretion in conditioned media from control and sFRP4-EGFP transfected U87MG cells. Data are shown as mean ± SEM. * *p* < 0.05, *** *p* < 0.0001, *n* = 3. (**C**) Photomicrographic representation of iPSCs, iPSC-GBM, iPSC-GSCs and iPSCs+U87MG sFRP4 CM, scale bar = 100 μm. (**D**) qRT-PCR analysis of sFRP4 in iPSCs, iPSC-GBM, iPSC-GSCs and iPSCs+U87MG sFRP4 CM. Data are shown as mean ± SEM. ** *p* < 0.001. (**E**) qRT-PCR analysis of MGMT in iPSC-GBM and iPSCs+U87MG sFRP4 CM. Data are shown as mean ± SEM. * *p* < 0.05. (**F**) qRT-PCR analysis of CD133, LEF1, β-catenin and Wnt3a in iPSC-GSCs and iPSCs+U87MG sFRP4 CM. Data are shown as mean ± SEM. * *p* < 0.05, ** *p* < 0.001, *** *p* < 0.0001, *n* = 3. (**G**) Co-immunostaining of β-catenin and CD44 in iPSC-GSCs and sFRP4 protein treated iPSC-GSCs, scale bar = 100 μm. Arrow indicates nuclear localization of β-catenin.

As upregulation of the Wnt pathway was observed in GBM and GSCs, iPSCs were induced with U87MG sFRP4 CM to understand if sFRP4 overexpression reverses the conversion process. Therefore, first, we transiently transfected U87MG with sFRP4-EGFP, and transient transfection was confirmed using fluorescence microscopy (Figure 6A). Furthermore, indirect ELISA demonstrated that sFRP4 was overexpressed in cell lysate from sFRP4-transfected U87MG. In addition, indirect ELISA also validated that sFRP4 was secreted in conditioned media (Figure 6B). Inducing iPSC with sFRP4 secreted CM did not show any morphological changes, and neuron-like expansion was not found in these cells, as seen with iPSC-GBM (Figure 6C). sFRP4 expression was then investigated in iPSC, iPSC-GBM, iPSC-GSCs, iPSC-derived sFRP4 overexpressed glioblastoma cells and was found to be upregulated only in iPSC-derived sFRP4 overexpressed glioblastoma cells as confirmed by qPCR (Figure 6D).

Furthermore, we compared iPSC-GBM with iPSC-derived sFRP4 overexpressed glioblastoma cells and found MGMT expression was downregulated in iPSC-derived sFRP4 overexpressed glioblastoma cells (Figure 6E). In addition, LEF1, β-catenin and Wnt3a as well as CD133 were found to be downregulated in iPSC-derived sFRP4 overexpressed glioblastoma cells compared to iPSC-GSCs (Figure 6F). These results indicate that sFRP4 overexpression reverses the conversion of iPSC into GBM and GSCs by decreasing the Wnt/β-catenin pathway. Furthermore, we also evaluated the iPSC-GSCs model with recombinant human sFRP4 protein, and co-immunostaining revealed that sFRP4 decreases nuclear localization of β-catenin and decreases expression of CD44 in iPSC-GSCs (Figure 6G). This further confirms that the iPSC-derived GSC model could be used for the screening of chemotherapeutic drugs.

## 4. Discussion

iPSCs have gained increased attention in regenerative medicine and disease modelling. The creation of the CSCs model from iPSCs has ushered in a new avenue for understanding the molecular landscape of CSCs. Treatment failure for GBM results because of lacuna in understanding the mechanisms of disease progression. In vitro or animal models fail to recapitulate the diseased complexity [52]. Therefore, we attempted to establish an iPSC-derived GBM model which would help in understanding the molecular mechanisms of disease progression.

iPSCs can be generated from any somatic cell types, such as fibroblasts, cord blood and peripheral blood, either by integrative or non-integrative reprogramming methods. Pipino et al. 2013 discussed the advantages of using the placenta as a promising source of stem cells over embryonic stem cells and adult stem cells [53]. In addition, placental stem cells sustain immunosuppressive properties in vivo [53]. Furthermore, our group reported and described AMMSCs as a storehouse of stem cells [27]. Reprogramming of human fetal chorionic mesenchymal stromal cells extracted from term pregnancies by Yamanaka factors has proven to be a promising source for the generation of iPSCs [54]. Furthermore, generation of iPSCs from umbilical cord matrix and AMMSCs by transduction using pMX-based retroviruses and mitomycin C-inactivated mouse embryonic fibroblasts as feeders have been reported [28]. It is worthwhile to observe that these generated iPSCs are exempt from the abnormalities associated with iPSCs derived from other sources [28]. Therefore, in this present study, we reprogrammed AMMSCs using integration-free episomal plasmids for ectopic expression of Yamanaka factors. Typical iPSC morphology resembles that of ESCs with packed large nucleolus, minimal cytoplasm, and sharp edges [52,55]. iPSC colonies started appearing from day 10–12 of reprogramming with typical human ESC morphology. Muthusamy et al. 2014 reported cytoplasmic lipid bodies of iPSCs which in the epiblast state internalize retinol from the media, sequester it as retinyl ester, store it in lipid bodies and, during differentiation, oxidize it to retinoic acid, which fluoresces blue at 450–500 nm. These fluorescing lipid bodies differentiate human iPSCs from mESCs, as they are absent in mESCs but are present in pluripotent mouse epiblast-like cells. In this study, we demonstrated the presence of blue fluorescence, which indicates lipid bodies, further confirming the pluripotent state of iPSCs. In addition, live AP staining has been proven as a promising tool for detecting pluripotency. iPSC colonies were evaluated for expression of AP by live AP staining, and AP positive colonies were then selected and propagated further.

Furthermore, OCT3/4 and SOX2 synergistically act to accelerate the expression of stemness genes and decrease the expression of differentiating genes [3]. In addition, KLF4 plays a vital role in suppressing the somatic genes in the early stage and activation of pluripotency genes in the later stage during reprogramming [56,57]. Furthermore, c-Myc could act as a booster for reprogramming as described by Takahashi et al. 2007 [3]. A typical negative marker for iPSCs is the glycoprotein CD44, as it is absent in iPSCs [58]. Another negative marker for iPSCs is vimentin which, during reprogramming from somatic cells, undergoes mesenchymal-to-epithelial transition and is a well-known mesenchymal marker [59,60,61,62]. In the present study, qPCR and immunofluorescence staining confirmed overexpression of OCT3/4, KLF4, c-Myc, NANOG and ESC-specific surface markers [3,63] SSEA-4, tumor-related antigen TRA-1-81, and TRA-1-60 and decreased expression of CD44 and vimentin in iPSCs compared to its parental cells, AMMSCs. From these observations, we could assume efficient conversion of AMMSCs induced to iPSCs which was then further confirmed for germ layers’ differentiation ability.

Pluripotency of hESCs is sustained by activin/nodal signaling [64]. In the present study, we induced iPSCs differentiation into neuroectoderm by activin/nodal receptor kinase (ALK/4/5/7) inhibitor SB431542 [31], and then, neuronal differentiation and survival was induced by providing N-2 supplement and B-27, respectively. PAX6, a key factor, is required in human neuroectoderm specification from human ESCs [65]. Immunocytochemical staining demonstrated PAX6-positive neuroectoderm differentiated from iPSCs.

In the present study, we approached in vitro cardiac mesoderm differentiation from iPSCs by following a GiWi protocol, where first Wnt activation is achieved by a GSK3β inhibitor CHIR99021 (which is the Gi step) to induce meso-endoderm-specific genes and to inhibit ectoderm differentiation. Mesoderm-specific genes were then activated by maintaining cells without CHIR99021. Differentiation of mesoderm cells into cardiomyocyte lineage was achieved by inhibition of the Wnt pathway by IWR-1 (which is the Wi step). In addition, apoptosis and oxidative stress generated in these steps are reduced by addition of insulin, which enhances cell survival and proliferation. Furthermore, ascorbic acid and SB431542 were used to enhance cardiac differentiation [33,66,67,68,69]. Nkx2.5, a pivotal transcription factor, specifies heart formation in mesoderm [70]. Immunocytochemical staining revealed that Nkx2.5-positive cardiac mesoderm was generated from iPSCs.

Generation of pancreatic β -cells from human iPS cells has gained tremendous interest in the treatment of type I diabetes [39]. In the present study, we first activated the Wnt/β-catenin pathway by CHIR99021 to induce meso-endoderm. Then TGF- β pathway was activated at a low concentration of activin to differentiate into definitive endoderm. Differentiation into pancreatic progenitor cells was then enhanced by TGF-β inhibition using SB431542 in combination with retinoic acid, nicotinamide and EGF. Retinoic acid is required for generating insulin-expressing β cells. In addition, nicotinamide is used to induce differentiation into pancreatic lineage by inhibiting casein kinase I (CKI) and ROCK kinase pathways. Furthermore, EGF plays a pivotal role in pancreatic lineage cell expansion, maturation and specification into pancreatic endoderm cells followed by insulin-producing β cells [39,71,72,73,74,75,76]. The generated pancreatic progenitor cells were positively stained for DTZ. Therefore, our small molecules approach for differentiation of iPSC enabled efficient generation of insulin-producing β cells, which confirms that these generated iPSC can also successfully differentiate into endoderm lineage. From these observations, we could demonstrate that these iPSCs generated from perinatal sources can efficiently differentiate into three germ layers.

Tumor originates either from radiation/mutation-induced transformed differentiated cells or from normal stem cells which had undergone oncogenic mutations [77]. CD34+CD38- CSCs were first recognized in acute myeloid leukemia [78]. Stem cells were first identified from the solid tumor as reported by the group of Al-Hajj [79]. Later, CD133 expressing CSCs was first identified in brain tumors by Singh et al. 2003, 2004 [80,81]. CSCs have a profound impact on metastasis [11], effectively resist chemotherapy and radiation by overexpressing ATP-binding cassettes (ABC) transporters like ABCC1, ABCG2 and aldehyde dehydrogenase markers (ALDH) [82,83]. Furthermore, the self-renewal capacity of CSCs is regulated by the NF-κB pathway, JAK-STAT pathway, TGF/SMAD pathway, and PI3K/AKT/mTOR pathway [11]. In addition, Notch, TGF-β, Wnt, AKT, and EGFR pathways drive drug resistance of glioblastoma stem cells (GSCs) [84]. Furthermore, the Wnt/β-catenin pathway promotes self-renewal of CSCs by activating the downstream enhancer of zeste homolog 2 (EZH2) [85] and by upregulating expression of cyclin D1, cyclin E, which converts dormant CSCs into an active state [11,86]. Although several signal transduction pathways are involved in regulating CSCs, the principal pathway responsible for the disease progression of GBM and the origin of CSCs remains to be identified. Therefore, we established a rapid and easy iPSC-derived glioblastoma and glioblastoma stem cells (GSCs) models which would be an efficient platform for understanding and analyzing molecular mechanisms of disease progression.

The tumor microenvironment (TME) consists of stem cells, differentiated cells, extracellular matrix, mesenchymal stem cells, cancer-associated fibroblasts, endothelial cells, immune system cells, cytokines, and growth factors [87]. TME enhances the tumor survival pathway by secretion of fibroblast growth factor, hepatocyte growth factor, interleukin-6, and transforming growth factor-β [88,89]. CSCs interact with TME by secreting VEGF, MMPs, TGF-β, HIF 1 and miRNA enclosed in exosome or microvesicles and have a profound impact on cancer development and progression [89]. Glioma CSCs maintain their self-renewal in an autocrine TGF-β-dependent manner [90]. Afify et al. 2019 reported the protocol of conversion of mouse iPSCs/ESCs into CSCs using conditioned media (CM) derived from Lewis lung carcinoma cell line LL/2(LLC1), breast cancer cell line T47D, liver cancer cell line PLC/PRF/5, hepatocellular carcinoma cell line (Huh7) and pancreatic carcinoma cell line PK-8 and KLM-1 [1]. The derived CSCs could induce benign teratoma, angiogenesis, exhibit expression of Ki67, N-cadherin, NANOG, KLF4, c-Myc and stemness markers CD24, CD90, and CD44 [1,21]. In addition, a recent study reported that extracellular vesicles (EV) derived from the Lewis lung carcinoma (LLC) cell line could endow normal stem cells with CSC-like properties, and these cells could induce malignant liposarcomas and angiogenesis in vivo [91]. Conditioned media (CM) from cancer cells contains various inflammatory cytokines, chemokines, and extracellular matrix components which activates signal transduction pathways and maintains cellular alterations and chromosomal instability [21,92,93,94]. In addition, chronic microenvironment induces cells to mimic cancer like phenotypes. Therefore, the CM induced iPSC-derived CSC model has gained increased attention. We followed a similar approach and successfully converted iPSC into GBM and GSCs using CM. It is also possible that some of the pro-tumorigenic factors, c-Myc, TRA-1-60, and TRA-1-81, expressed in derived iPSCs, probably accelerated the conversion.

O^6^-methylguanine DNA methyltransferase (MGMT) is an enzyme that confers glioblastoma with chemotherapeutic resistance [95]. In addition, crosstalk between glioma-associated oncogene GLI2 and the Wnt/β-catenin pathway drives cell invasion, and GLI2 acts as a regulator of β-catenin [96]. In our study, there was a tendency for MGMT overexpression in iPSCs induced with a U87MG monolayer CM and GLI2 was found to be over expressed, which confirmed these as iPSC-derived glioblastoma-like cells. In addition, CSCs CM induced iPSCs with over-expression of CSC-specific markers CD133, CD44, ABCG2, and ABCC2, confirming their conversion to GSCs. All these derived models were made successfully after a conversion period of five days using only CM. Our study demonstrated a faster and easier approach of generating a CSC model of human origin in five days using CM without use of any additional compound. Earlier, it has been shown that miPSC was successfully converted into CSCs in one week with the use of inhibitors of signal transduction [97]. Furthermore, Hassan et al. 2022 demonstrated that different pancreatic cell lines induce miPSCs into CSCs with different gene expression and plasticity [98]. Taking these observations into consideration, we expect that this model could resemble the heterogeneity of GBM in terms of different cell types. The heterogeneity of the iPSC-derived GBM cells can be further confirmed by creating an in vivo GBM model.

In the present study, we also observed a tendency of increased expression of a lymphoid enhancer-binding factor 1 (LEF1) in iPSC-derived glioblastoma and iPSCs-derived glioblastoma stem cells. Previously, it was reported that LEF1 drives stemness in esophageal squamous cell carcinoma by upregulating the TGF-β pathway [99] and also facilitating EMT [100]. LEF-1 plays a role in the metastasis of GBM [100] and upregulates the expression of CD133 and nestin [101]. The tendency of increased expression of LEF1 in our study could also be instrumental in upregulating stemness marker CD133 in GBM.

Glioma cell proliferation and invasion are maintained by β-catenin/TCF4 activity [102]. Expectedly, we observed increased β-catenin and a tendency of Wnt3a expression in iPSC-derived models. Wnt3a contributes to the chemoresistance of glioma-derived stem-like cells [103]. In addition, immunofluorescence staining also revealed that iPSC-derived glioblastoma cells have upregulated nuclear expression of β-catenin compared to iPSCs, which is a hallmark of the activated Wnt/β-catenin pathway. Taking these observations into consideration, upregulation of the Wnt/β-catenin pathway was implied to be associated with the stemness of iPSC-derived glioblastoma cells.

Our previous study reported that the netrin-like domain of Wnt antagonist sFRP4 inhibits the stemness of GBM in vitro [26]. Therefore, we investigated if sFRP4 overexpression reverses the conversion of iPSC into glioblastoma cells. Expectedly, overexpression of sFRP4 was associated with downregulation of LEF1, WNT3a, β-catenin, MGMT, and CD133. This may indicate that the Wnt pathway regulates glioma-specific markers in iPSC-derived glioma cells, and conversion was reversed with overexpression of sFRP4. We also evaluated our iPSC-derived GSCs model with sFRP4 protein and we found that sFRP4 decreases nuclear expression of β-catenin and the GSC marker CD44. This clearly indicates that our generated iPSC-derived glioblastoma model could be used for analyzing and screening chemotherapeutic drugs.

## 5. Conclusions

Our study has demonstrated the creation of rapid and easy models of GBM and cancer stem cell models from iPSCs derived from an easily available stem cell source. This hiPSC-derived model would be an efficient platform for analyzing multiple signal transduction pathways. In addition, this would also help in screening of chemotherapeutic drugs for GBM and CSCs. This study has also opened an avenue for understanding the molecular landscape of GBM. Furthermore, we also observed upregulation of the canonical Wnt/β-catenin signal transduction which is a prominent feature of GBM. In addition, the Wnt antagonist, sFRP4, suppresses features of GBM. We demonstrated the creation of a novel platform wherein iPSC can be easily derived from perinatal stem cells and can be rapidly converted to GBM stem cells which can be further used for developing drug targets.

## Figures and Tables

**Table 1:** List of genes and their primer sequences.

Gene	Sequences 5′-3′	Product Length (bp)	Annealing Temperature (°C)
GAPDH	FP: CGACCACTTGTCAAGCTCA RP: AGGGGAGATTCAGTGTGGT	202	60 °C
OCT3/4	FP: GAAGGATGTGGTCCGAGTGT RP: TTGTGTTCCCAATTCCTTCC	400	60 °C
KLF4	FP: GGCGAGAAACCTTACCACTGT RP: TACTGAACTCTCTCTGCTGGCA	370, 454, 622, 706, 1042, 538, 874, 958	60 °C
c-Myc	FP: GCGTCCTGGGAAGGGAGATCCGGAGC RP: TTGAGGGGCATCGTCGCGGGAGGCTG	328,325	60 °C
NANOG	FP: TTTGTGGGCCTGAAGAAAACT RP: AGGGCTGTCCTGAATAAGCAG	116	60 °C
CD44	FP: CATCTACCCCAGCAACCCTA RP: GGTTGTGTTTGCTCCACCTT	271	60 °C
CD133 (PROM1)	FP: TCAGTGAGAAAGTGGCATCG RP: TGTTGTGATGGGCTTGTCAT	313, 244	60 °C
ABCG2	FP: GTGGCATTAAACAGAGAAGAAGACT RP: CACCCCCGGAAAGTTGATGT	148	60 °C
ABCC2	FP: AGAGCTGGCCCTTGTACTCA RP: TGCGTTTCAAACTTGCTCAC	492	60 °C
MGMT	FP: CACCGTTTGCGACTTGGTACTT RP: AGACCCTGCTCACAACCAGACA	111	58 °C
GLI2	FP: CATGGAGCACTACCTCCGTTC RP: CGACGGTCATCTGGTGGTAAT	173	60 °C
LEF1	FP: AAATGGGTCCCTTTCTCCAC RP: TCGTCGCTGTAGGTGATGAG	108	59 °C
β-catenin	FP: CGTCCACAACACTCTGGCTA RP: GCCAGCACTTCACTGCAATA	159	55 °C
Wnt3a	FP: ACTACGTGGAGATCATGCCC RP: ATGAGCGTGTCACTGCAAAG	210	54 °C
sFRP4	FP: CGATCGGTGCAAGTGTAAA RP: GACTTGAGTTCGAGGGATGG	181	49 °C

## Data Availability

The datasets used and/or analyzed during the current study are available from the corresponding author on reasonable request.

## Supplementary Materials

The following supporting information can be downloaded at: https://www.mdpi.com/article/10.3390/cancers15143622/s1. Details of materials and antibodies are listed in supplementary information. Table S1: List of chemicals. Table S2: List of antibodies.

## Abbreviations

AA: Ascorbic acid; AMMSCs: Amniotic membrane-derived mesenchymal stem cells; AP: Alkaline phosphatase; BSA: Bovine serum albumin; CiRA: Center for iPS Cell Research and Application; CK1:Casein kinase I; CM: Conditioned Media; CSCs: Cancer stem cells; DAPI: 4’,6-Diamidino-2-phenylindole dihydrochloride; DMEM: Dulbecco’s Modified Eagle Medium; DTZ: Diphenyl thiocarbazone or dithizone; EGF: Epidermal growth factor; ESCs: Embryonic Stem Cells; FBS: Fetal Bovine Serum; FGF2: Fibroblast growth factor 2; GBM: Glioblastoma multiforme; GLI2: Glioma-associated oncogene 2; iPSCs: Induced pluripotent stem cells; ITS: insulin transferrin selenite; LEF1:Lymphoid enhancer binding factor 1; MGMT: Methyl guanine methyltransferase; PAX6: Paired Box Gene 6; PBS: Phosphate buffered saline; PFA: Paraformaldehyde; PMSF: phenylmethanesulfonylfluoride; qPCR: Quantitative real-time polymerase chain reaction; RPMI: Rosewell park memorial institute medium; sFRP4: secreted frizzled-related protein 4; SSEA4: Stage-Specific Embryonic Antigen-4; TRA: Tumor-related antigen; NEAA: Non-essential amino acids.
