# Supplementary material for: iPSC-Derived Glioblastoma Cells Have Enhanced Stemness Wnt/β-Catenin Activity Which Is Negatively Regulated by Wnt Antagonist sFRP4
Source: Cancers (Basel). 2023 Jul 14;15(14):3622. doi: 10.3390/cancers15143622 (PMC10377620; doi:10.3390/cancers15143622)
Supplement: Supplementary file 1 [file cancers-15-03622-s001.zip › cancers-2387006-Supplementary.pdf]

Supplementary Information

Supplementary Tables

Table S1: List of chemicals

Chemicals	Catalogue	Company
DMEM	Cat#110995065	Gibco ™, Thermo Fisher Scientific, US
FBS	Cat#10270106	Gibco ™, Thermo Fisher Scientific, US
Antibiotic solution	Cat#A001	Himedia Laboratories Pvt. Ltd, Bangalore, India
pCXLE-hOCT3/4-shp53-F<br>pCXLE-Hsk<br>pCXLE-hUL	Cat# 27077, 27078, 27080	Addgene, Watertown, Massachusetts, United States (US)
Lipofectamine™3000	Cat# L3000008	Invitrogen, Waltham, Massachusetts, US
Knockout ™ DMEM 1X, optimised for ES cells	Cat#10-829-018	Gibco ™, Thermo Fisher Scientific, US
Knockout ™ Serum replacement	Cat#10828028	Gibco ™, Thermo Fisher Scientific, US
GlutaMAX ™ supplement	Cat#35-050-061	Gibco ™, Thermo Fisher Scientific, US
NEAA	Cat#11140050	Gibco ™, Thermo Fisher Scientific, US
2-mercaptoethanol	Cat#M3148	Sigma-Aldrich, St. Louis, Missouri, US
FGF2 protein, human recombinant	Cat#10014-HNAE	Sino Biological, China
Matrigel	Cat# 356230	Corning, New York, US
DMEM/F-12	Cat#11320033	Gibco ™, Thermo Fisher Scientific, US
Alkaline Phosphatase Live Stain	Cat#A14353	Invitrogen, Waltham, Massachusetts, US
RNAiso Plus	Cat# 9108	TaKaRa Bio INC, Shiga, Japan
PrimeScript 1st strand cDNA synthesis kit	Cat# 6110A	TaKaRa Bio INC, Shiga, Japan
TB Green® Premix Ex Taq™ II	Cat# RR820A	TaKaRa Bio INC, Shiga, Japan
Hoechst 33342, Trihydrochloride, Trihydrate solution in water	Cat# H3570	Thermo Fisher Scientific, Waltham, Massachusetts, US
DAPI	Cat#PCT1539	Himedia Laboratories Pvt. Ltd, Bangalore, India
SB431542 hydrate	Cat#S4317	Sigma-Aldrich, St. Louis, Missouri, US
N-2 supplement(100X)	Cat#17502048	Gibco ™, Thermo Fisher Scientific, US

B-27™ Supplement (50X), serum free	Cat#17504044	Gibco ™, Thermo Fisher Scientific, US
RPMI 1640, 1X	Cat#A1049101	Himedia Laboratories Pvt. Ltd, Bangalore, India
IWR-1	Cat#I0161	Sigma-Aldrich, St. Louis, MO, US
Ascorbic Acid	Cat# 23006(0149100)	SRL Research Laboratories Pvt.Ltd., India
Insulin solution from bovine pancreases	Cat#I0516	Sigma-Aldrich, St. Louis, MO, US
ITS liquid media supplement	Cat#I3146	Sigma-Aldrich, St. Louis, MO, US
Wnt 3a human	Cat#CYT-861	Prospec Bio, Rehovot, Israel
CHIR99021	Cat#SML1046	Sigma-Aldrich, St. Louis, MO, US
Activin A	Cat#A4941	Sigma-Aldrich, St. Louis, MO, US
Nicotinamide	Cat#N0636	Sigma-Aldrich, St. Louis, MO, US
Retinoic Acid	Cat#R2625	Sigma-Aldrich, St. Louis, MO, US
EGF	Cat#01-107	Millipore, Burlington, Massachusetts, US
DTZ	Cat#43820	Sigma-Aldrich, St. Louis, MO, US
PFA	Cat#TC703	Himedia Laboratories Pvt. Ltd, Bangalore, India
PBS	Cat#P4417	Sigma-Aldrich, St. Louis, MO, US
BSA	Cat#MB083	Himedia Laboratories Pvt. Ltd, Bangalore, India
RIPA Buffer	Cat#R0278	Sigma-Aldrich, St. Louis, MO, US
Phenylmethanesulfonylfluoride	Cat#P7626	Sigma-Aldrich, St. Louis, MO, US
3,3’,5,5’-Tetramethylbenzidine (TMB)Liquid Substrate system for Membranes	Cat#T0565	Sigma-Aldrich, St. Louis, MO, US
Complete EDTA-free Protease Inhibitor Cocktail	Cat#4693132001	Roche, Basel, Switzerland
Recombinant Human sFRP-4 Protein	Cat#1827-SF-025/CF	R & D Systems, Inc., Minneapolis, MN 55413, USA

Table S2: List of antibodies

Antibody	Catalouge	Company
Mouse anti-Oct-4 antibody	Cat# MAB4305	Sigma-Aldrich, St. Louis, Missouri, US
Purified mouse anti-Sox2 antibody clone 030-678	Cat# 561469	BD Biosciences, US
Mouse anti-Stage-Specific Embryonic Antigen-4 (SSEA-4) antibody, clone MC-813-70	Cat# MAB4304	Sigma-Aldrich, St. Louis, Missouri, US
Purified mouse anti-human TRA-1-60 antigen	Cat# 560071	BD Pharmingen ™, US
Mouse anti-TRA-1-81	Cat#MAB4381	Sigma-Aldrich, St. Louis, Missouri, US
Rabbit anti-Vimentin	Cat# CUSABPAA4450	Cusabio Technology LLC, Houston, Texas
Rabbit anti- Paired Box Gene 6 (PAX6)	Cat# PAH446Ra01	Cloud-Clone Corp., Houston, US
Rabbit anti-Nkx2.5 polyclonal antibody	Cat#PA5-49431	Thermo Fisher Scientific, Waltham, Massachusetts, US
Mouse CD133 monoclonal antibody FITC, Clone: EMK08	Cat#11-133-942	eBioscience™ , Thermo Fisher Scientific, Waltham, Massachusetts, US
Mouse CD44 monoclonal antibody (IM7), FITC,	Cat#11-0441-82	eBioscience™, Invitrogen, Waltham, Massachusetts, US
Rabbit β-catenin polyclonal antibody	Cat#PA5-16762	Thermo Fisher Scientific, Waltham, Massachusetts, US
Goat anti-mouse IgG (H+L) highly cross-adsorbed secondary antibody, Alexa Fluor™ 594	Cat# A-11032	Invitrogen, Waltham, Massachusetts, US
Goat anti-rabbit IgG (H+L) highly cross-adsorbed secondary antibody, Alexa Fluor™ 488	Cat#A32731	Invitrogen, Waltham, Massachusetts, US
Goat anti-rabbit IgG(H+L) cross-adsorbed secondary antibody, Alexa Fluor ™ 594	Cat# A-11012	Thermo Fisher Scientific, Waltham, Massachusetts, US
FITC Rat IgG 2b, κ isotype control antibody	Cat#400633	BioLegend, San Diego, CA, US
Rabbit sFRP4 polyclonal antibody	Cat#15328-1-AP	Proteintech Group, USA
Goat anti-Rabbit IgG(H+L) Secondary Antibody,HRP	Cat#32460	Thermo Fisher Scientific, Waltham, Massachusetts, US
